# Methylglyoxal mildly impairs spatial memory and reduces expression of excitatory synaptic genes in the hippocampus of male mice

**DOI:** 10.14814/phy2.71077

**Published:** 2026-08-21

**Authors:** Akane Nakao, Takumi Yokokawa, Fuminori Okazaki, Nobumasa Iwanaka, Kazuo Inoue, Tatsuya Hayashi, Tatsuro Egawa

**Affiliations:** ^1^ Division of Food Science and Biotechnology, Graduate School of Agriculture Kyoto University Kyoto Japan; ^2^ Faculty of Health Science Kyoto Koka University Kyoto Japan; ^3^ Laboratory of Sports and Exercise Medicine, Graduate School of Human and Environmental Studies Kyoto University Kyoto Japan; ^4^ Laboratory of Health and Exercise Sciences, Graduate School of Human and Environmental Studies Kyoto University Kyoto Japan

**Keywords:** hippocampus, memory, metabolism, methylglyoxal, synapse

## Abstract

Diabetes is associated with cognitive impairment and alterations in synaptic molecules; however, the factors within the diabetic milieu that drive these central changes remain unclear. Methylglyoxal (MG), a reactive dicarbonyl and major precursor of advanced glycation end products, accumulates under hyperglycemic and metabolically compromised conditions and is a candidate mediator of diabetic phenotypes. Here, we examined whether chronic MG exposure affects metabolism, behavior, and the expression of synaptic genes in the hippocampus and prefrontal cortex. Mice received MG in drinking water for 1 or 3 months, and then metabolic, behavioral, and biochemical analyses were performed. MG mildly impaired glucose clearance and induced insulin resistance, together with reduced adiposity in the 3‐month intervention, indicating a disturbance of glucose and lipid metabolism. MG did not consistently affect anxiety‐like behavior. In the Barnes maze test, MG mildly impaired spatial memory performance. In the hippocampus, MG selectively decreased the expression levels of the excitatory synaptic genes, whereas no gene was altered in the prefrontal cortex. These findings identify learning‐associated excitatory synaptic genes as targets of chronic MG exposure and position MG as a candidate factor linking the diabetic metabolic state to hippocampal synaptic and cognitive alterations.

## 1 INTRODUCTION

Beyond its peripheral metabolic disturbances, diabetes mellitus is also associated with an increased risk of cognitive impairment and psychiatric disorders such as depression and anxiety (Gispen & Biessels, 2000; Golden et al., 2008; Smith et al., 2013). In both type 1 and type 2 diabetes, patients and animal models exhibit deficits in learning and memory, as well as alterations in synaptic plasticity (Biessels et al., 1996; Gispen & Biessels, 2000; Stranahan et al., 2008). Together, these observations suggest that peripheral metabolic dysregulation can extend to central synaptic function. However, the molecular mechanisms linking the diabetic metabolic state to these synaptic changes remain incompletely understood.

Synaptic function depends on a coordinated series of molecular processes at both excitatory and inhibitory synapses: the loading of neurotransmitters into presynaptic vesicles, the clustering and stabilization of receptors by postsynaptic scaffolding proteins, and receptor‐mediated transmission and plasticity. Altered expression of these molecules can compromise synaptic function, cognition, and emotional behavior: changes in excitatory synaptic transmission and plasticity are closely related to learning and memory (Huganir & Nicoll, 2013), whereas disruption of inhibitory signaling and the excitatory‐inhibitory balance is linked to anxiety‐ and depression‐like behavior (Duman et al., 2019). We previously reported that type 1 diabetic mice show a partial decrease in the expression of excitatory and inhibitory synaptic proteins in the hippocampus and cortex (Yokokawa et al., 2023), indicating that changes in synaptic molecule expression accompany the progression of diabetes. Nevertheless, among the many constituents of the diabetic milieu, such as hyperglycemia, insulin deficiency, oxidative stress, and the accumulation of glycation products, it remains unclear which factor drives these synaptic changes.

One candidate factor is methylglyoxal (MG), a highly reactive dicarbonyl compound generated endogenously as a by‐product of glycolysis. Under hyperglycemic and metabolically compromised conditions, MG accumulates and acts as a major precursor of advanced glycation end products (AGEs), irreversibly modified proteins that accumulate in tissues and can trigger cellular signaling (Schalkwijk & Stehouwer, 2020). With respect to its actions in the brain, in vivo MG administration impairs learning and memory and induces neuroinflammation and oxidative stress mediated by the receptor for AGEs (RAGE) (Pucci et al., 2021), as well as tau pathology (Samsuzzaman et al., 2024), while repeated administration additionally depletes prefrontal dopamine and produces memory impairment and depressive‐like behavior (Szczepanik et al., 2020). At the cellular level, exposure of cultured neurons to high concentrations of MG shortens the axon initial segment and alters neuronal excitability and network activity (Griggs et al., 2021). However, these studies have focused primarily on inflammatory, oxidative, neurodegenerative, neurotransmitter, or axonal mechanisms. Consequently, whether MG exposure alters the expression of the molecular constituents of the synapse remains unexamined.

Therefore, in the present study we examined whether chronic MG exposure affects metabolism, behavior, and the expression of genes encoding synaptic molecules in the hippocampus and prefrontal cortex. MG was administered in the drinking water for 1 or 3 months, after which we assessed metabolic indices including glucose tolerance and insulin sensitivity, anxiety‐like behavior, and spatial learning, and the hippocampal and prefrontal mRNA expression of synaptic genes. Importantly, the metabolic assessment also served to verify that the exposure paradigm was biologically effective. The aim was to determine whether MG exposure, in the absence of sustained hyperglycemia, contributes to the synaptic and cognitive alterations observed in diabetes.

## 2 METHODS

### 2.1 Animals and treatment

C57BL/6J mice were purchased from Shimizu Laboratory Supplies (Kyoto, Japan). Only male mice were used in this study. This design was chosen to avoid introducing female odor cues into the behavioral testing environment, which can alter anxiety‐related and exploratory behavior in male mice, and to avoid potential variability associated with the estrous cycle. Mice were housed at 23°C ± 2°C under a 12‐h light–dark cycle (lights on 06:00–18:00 h) and provided standard laboratory chow (MF; Oriental Yeast, Tokyo, Japan) and water ad libitum, unless otherwise noted. At 4 weeks of age, mice were randomly assigned to control and MG‐treated groups in the 1‐month and 3‐month interventions. MG (Sigma‐Aldrich, M0252) was dissolved in drinking water at a final concentration of 1% (w/v). MG‐containing water was provided ad libitum. Treatment was continued for 1 or 3 months. Body weight and food intake were monitored weekly. No formal a priori power calculation was performed. Sample sizes were determined based on preliminary experience from our research group's prior studies, using group sizes that reliably detect differences of comparable magnitude in similar assays. Final group sizes for each analysis, which varied slightly due to attrition or assay‐specific exclusions, are reported in the corresponding figure legends.

### 2.2 Intraperitoneal glucose tolerance test (IPGTT)

After a 16‐h fast, mice received an intraperitoneal injection of glucose (1 g/kg body weight). Blood was sampled from the tail vein, and blood glucose was measured at 0, 15, 30, 60, and 120 min. The area under the curve (AUC) was calculated and expressed relative to the control group.

### 2.3 Insulin tolerance test (ITT)

After a 2‐h fast, mice received an intraperitoneal injection of human insulin (Humulin R, Eli Lilly Japan, Kobe, Japan, 4987‐428‐02101‐4) at 0.75 U/kg body weight. Blood glucose was measured from the tail vein at 0, 20, 40, and 60 min. The AUC was calculated and expressed relative to the control group.

### 2.4 Indirect calorimetry

Oxygen consumption and the respiratory exchange ratio (RER) were measured using an Arco2000 metabolic measurement system (ArcoSystem, Chiba, Japan), and spontaneous locomotor activity was recorded simultaneously with an infrared sensor (Neuroscience Inc., Tokyo, Japan). Mice were acclimated to the metabolic chambers for at least 24 h prior to the subsequent 24‐h data collection period, and parameters were analyzed separately for the light and dark phases.

### 2.5 Rectal temperature measurement

Rectal temperature was measured using a PTM1 portable temperature monitor and RET‐3 probe (Physitemp, Clifton, NJ).

### 2.6 Open field test

All behavioral tests were conducted between 07:00 and 11:00 h. Mice were allowed to move freely in a circular open field apparatus (50 cm in diameter) for 5 min. Each subject was placed individually in the center of the apparatus. The total distance and time spent in the center area were recorded and calculated using ANY‐maze software (Stoelting Co., Wood Dale, IL, USA).

### 2.7 Elevated plus maze test

The apparatus consisted of two open arms (5 × 30 cm) and enclosed arms of the same size, with a central square (5 × 5 cm). The enclosed arms were surrounded by 20‐cm high walls. The maze was elevated to a height of 50 cm above the floor. Arms of the same type were arranged opposite each other. Mice were placed in the central square of the maze, and then behavior was recorded for 5 min. The number of arm entries, time spent in the open arms, closed arms, and central area, and the total distance traveled were measured automatically using ANY‐maze software.

### 2.8 Y‐maze test

Spontaneous alternation was assessed using a Y‐maze. The maze contained three gray arms placed at 120° angles. Mice were placed at the distal end of one arm and allowed to freely explore the maze for 8 min. An alternation was defined as consecutive entries into three different arms (i.e., overlapping triplet sets of arm entries without repetition), and the percentage of spontaneous alternation was calculated as [number of alternations/(total arm entries −2)] × 100. Arm entries and the total distance traveled were recorded automatically using ANY‐maze software.

### 2.9 Barnes maze test

The Barnes maze test was conducted on a white circular surface, 1.0 m in diameter, with 12 holes equally spaced around the perimeter. The circular open field was elevated 50 cm from the floor. A black escape box, which had paper cage bedding on its bottom, was located under one of the holes. The hole above the escape box represented the target hole. The location of the target was consistent for a given mouse but randomized across mice. Two trials per day were conducted for 4 successive days. On Day 5, a probe trial was conducted for 60 s without the escape box. The movement of mice was tracked using ANY‐maze software. During training, the primary latency (time to the first visit to the target hole), primary distance (path length to the first visit to the target hole), and primary errors (number of non‐target holes visited before the first visit to the target hole) were recorded. In the probe trial, the time spent in each quadrant (target, left, right, and opposite) was measured.

### 2.10 Tissue collection and organ weights

At the end of the intervention, mice were euthanized by decapitation. The liver, interscapular brown adipose tissue (BAT), inguinal and gonadal white adipose tissue (iWAT and gWAT), and the gastrocnemius (GAS), plantaris (PLA), and soleus (SOL) muscles were dissected and weighed. The hippocampus and prefrontal cortex were dissected, snap‐frozen in liquid nitrogen, and stored at −80°C until biochemical analyses.

### 2.11 Blood analysis

Serum samples were collected and centrifuged at 2000 *× g* at 4°C for 10 min. Serum triglyceride (TG), nonesterified fatty acid (NEFA), cholesterol, and glucose were measured using LabAssay kits (Fujifilm Wako, Osaka, Japan; 291‐94,501 for TG, 299‐94,301 for NEFA, 293‐93,601 for cholesterol, and 291‐94,001 for glucose). All assays were performed in duplicate.

### 2.12 Quantitative real‐time PCR

Quantitative real‐time PCR was performed as described previously (Yokokawa et al., 2015). Total RNA was extracted using QIAzol Lysis Reagent (Qiagen, 79,306) and reverse‐transcribed into complementary DNA (cDNA) using ReverTra Ace qPCR RT Master Mix (Toyobo, FSQ‐201). The synthesized cDNA was appropriately diluted with EASY Dilution (Takara Bio, 9160). Gene expression levels were measured using specific primers (Table 1) and THUNDERBIRD Next SYBR qPCR Mix (Toyobo, QPX‐201). Target gene expression levels were normalized to the expression level of *Hprt*. The gene that was minimally affected by treatment was selected as the internal control. All assays were performed in duplicate.

**TABLE 1:** Primers used for real‐time PCR.

Gene		Sequence (5′–3′)
*Syp*	Forward	CAGTTCCGGGTGGTCAAGG
	Reverse	ACTCTCCGTCTTGTTGGCAC
*Slc17a7*	Forward	TTGTGGCTACCTCCACCCTAA
	Reverse	CAGCCGACTCCGTTCTAAGG
*Dlg4*	Forward	TGAGATCAGTCATAGCAGCTACT
	Reverse	CTTCCTCCCCTAGCAGGTCC
*Gria1*	Forward	AAAGGAGTGTACGCCATCTTTG
	Reverse	TGTCAACGGGAAAACTTGGAG
*Grin1*	Forward	ATGCACCTGCTGACATTCG
	Reverse	TATTGGCCTGGTTTACTGCCT
*Slc32a1*	Forward	ACCTCCGTGTCCAACAAGTC
	Reverse	CAAAGTCGAGATCGTCGCAGT
*Gphn*	Forward	CAACCACGACCATCAAATCCG
	Reverse	CCAACAAAGAAGGATCTTGGACA
*Gabra1*	Forward	CGACTGCTGGACGGTTATGAC
	Reverse	GTGACGAAAATGTCGGTCTTCA
*Hprt*	Forward	TCAGTCAACGGGGGACATAAA
	Reverse	GGGGCTGTACTGCTTAACCAG

### 2.13 Statistical analysis

All statistical analyses were performed using GraphPad Prism 10 (GraphPad Software, San Diego, CA, USA). Data are presented as mean ± SD. In dot‐plot panels, each dot represents an individual mouse, except for food intake, for which each dot represents the mean food intake per mouse calculated for each cage. Comparisons between two groups were performed using two‐tailed Welch's *t*‐tests. Data with two factors (treatment and intervention period) were analyzed by two‐way ANOVA. Time‐course data were analyzed by repeated‐measures two‐way ANOVA. Multiple comparisons were performed using Šídák's multiple comparisons test. Statistical significance was set at *p* < 0.05.

## 3 RESULTS

### 3.1 Chronic MG treatment mildly impaired glucose handling and reduced adiposity

**FIGURE 1:**
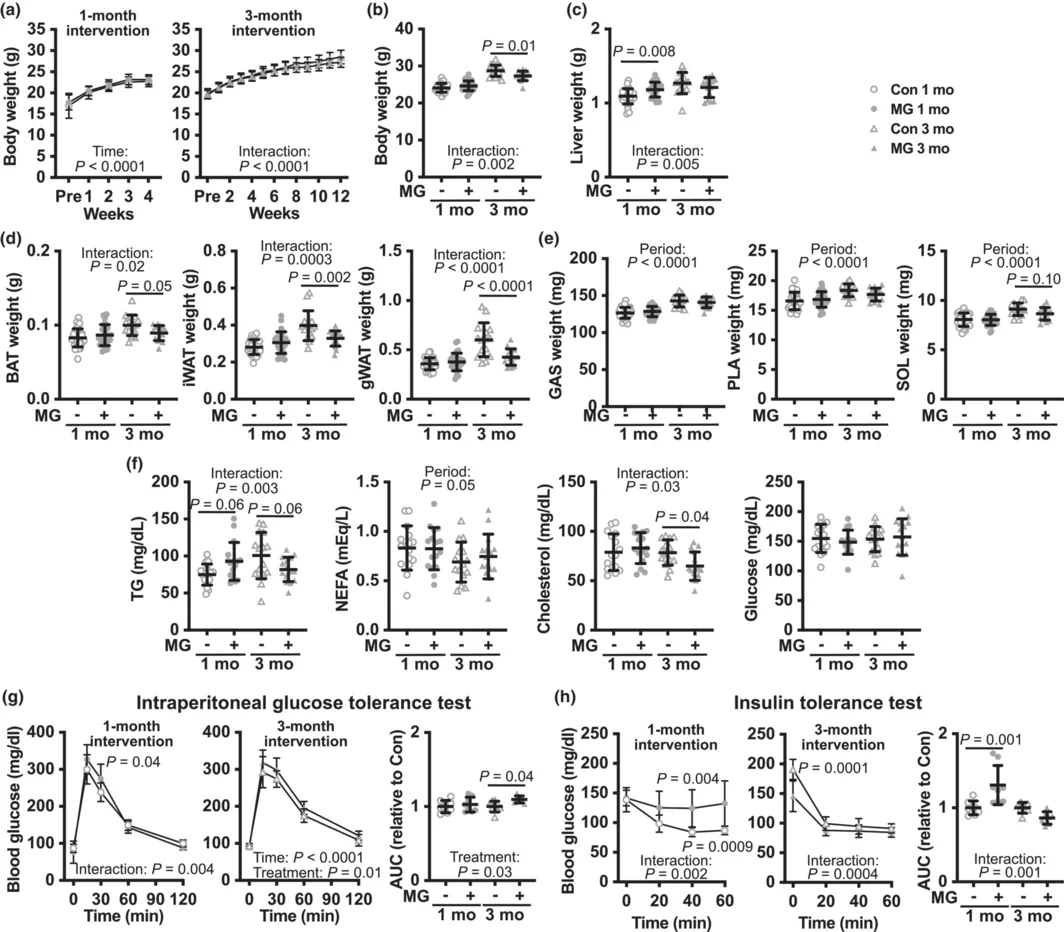
Metabolic effects of chronic MG treatment. (a) Body weight curves during the 1‐month and 3‐month interventions (*n* = 18–29 per group). (b) Body weight at dissection (*n* = 15–28 per group). (c) Wet weight of the liver (*n* = 15–28 per group). (d) Wet weights of brown adipose tissue (BAT), inguinal white adipose tissue (iWAT), and gonadal white adipose tissue (gWAT) (*n* = 15–28 per group). (e) Weights of the gastrocnemius (GAS), plantaris (PLA), and soleus (SOL) muscles (*n* = 15–28 per group). (f) Serum triglyceride (TG), nonesterified fatty acid (NEFA), cholesterol, and glucose levels in the fed state (*n* = 15–16 per group). (g) Intraperitoneal glucose tolerance test (IPGTT) in the 1‐month and 3‐month cohorts, and the area under the curve (AUC) expressed relative to control (*n* = 8 per group). (h) Insulin tolerance test (ITT) in the 1‐month and 3‐month interventions, and the AUC relative to control (*n* = 7–8 per group). Data are presented as mean ± SD.

Body weight increased over the intervention in both periods. In the 3‐month intervention, MG‐treated mice weighed less than controls at the end of the intervention, an effect not seen in the 1‐month intervention (Figure 1a,b). This lower body weight was accompanied by reduced fat mass: MG significantly decreased the wet weights of iWAT and gWAT in the 3‐month intervention, with brown adipose tissue showing a similar reduction that did not reach statistical significance (Figure 1d). Liver weight, in contrast, was significantly increased by MG in the 1‐month intervention with no difference at 3 months (Figure 1c). As expected from normal growth, skeletal muscle weights (GAS, PLA, SOL) were greater in the 3‐month than in the 1‐month intervention, and were unaffected by MG except for a reduction in the soleus at 3 months that did not reach statistical significance (Figure 1e).

Serum analyses revealed modest, time‐dependent metabolic shifts. In serum triglycerides, we found a significant treatment × period interaction and higher values with MG at 1 month and lower values at 3 months, although these differences did not reach statistical significance. Total cholesterol was significantly lower in MG mice in the 3‐month intervention, whereas NEFA and glucose were unchanged by MG treatment (Figure 1f). In the intraperitoneal glucose tolerance test, MG mildly impaired systemic glucose clearance in both interventions (Figure 1g). In the insulin tolerance test, MG produced clear insulin resistance at 1 month, while at 3 months MG significantly lowered basal glucose after a 2‐h fast (Figure 1h). Together, these data indicate that oral MG exerted reproducible, if modest, metabolic effects, confirming that the intervention was biologically effective.

### 3.2 MG reduced the RER but did not alter food intake or energy expenditure

**FIGURE 2:**
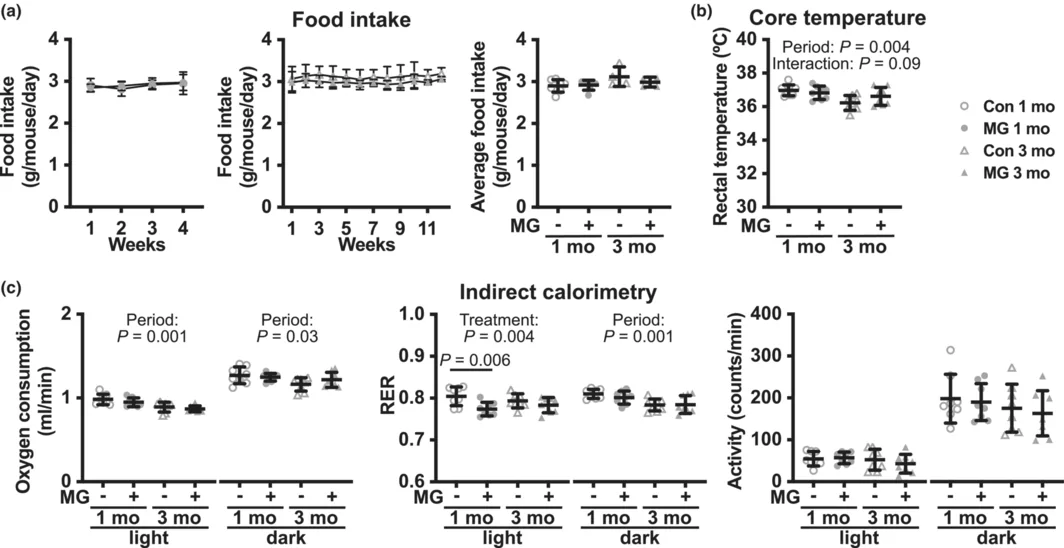
Food intake, rectal temperature, and indirect calorimetry in MG‐treated mice. (a) Daily food intake during the 1‐month and 3‐month interventions, and average food intake over the intervention (*n* = 4–7 cages per group). (b) Rectal temperature (*n* = 8 per group). (c) Oxygen consumption, respiratory exchange ratio (RER), and spontaneous locomotor activity in indirect calorimetry measured separately during the light and dark phases (*n* = 8–16 per group). Data are presented as mean ± SD.

Daily and average food intake were comparable between MG and control mice in both interventions (Figure 2a). Core body temperature differed only with intervention period, with no main effect of MG (Figure 2b). By indirect calorimetry, oxygen consumption and spontaneous locomotor activity were unaffected by MG, showing only the expected light/dark and period differences (Figure 2c). However, the RER was lowered by MG during the light phase, with a significant reduction in the 1‐month intervention on post hoc testing; the dark‐phase RER showed only the expected period difference (Figure 2c). Thus, although energy intake and whole‐body energy expenditure were preserved, MG elicited a modest shift toward fat oxidation.

### 3.3 MG did not consistently alter anxiety‐like behavior

**FIGURE 3:**
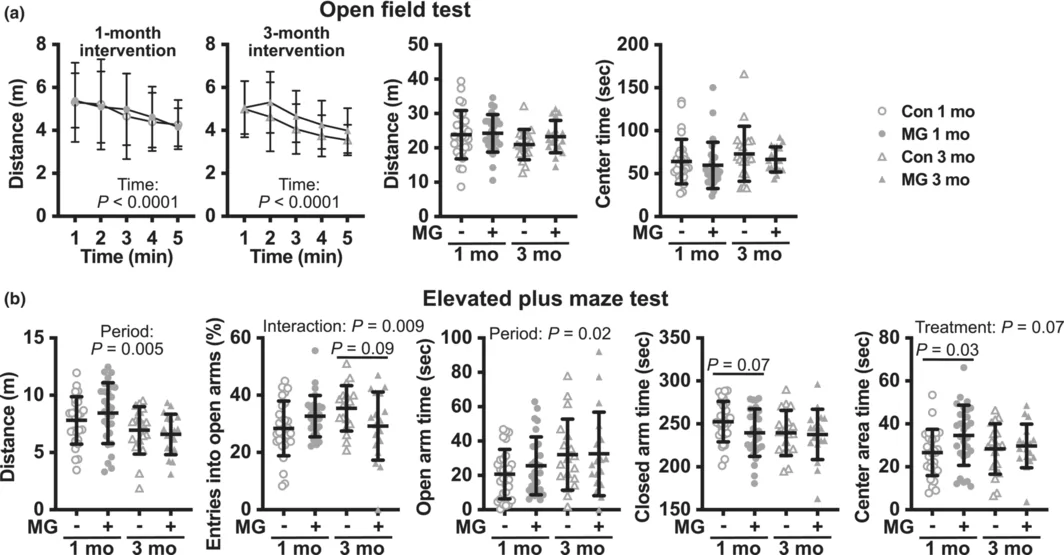
Open field and elevated plus maze behavior in MG‐treated mice. (a) Open field test: Distance traveled per minute during the 1‐month and 3‐month interventions, total distance traveled, and time spent in the center area (*n* = 17–29 per group). (b) Elevated plus maze test: Total distance traveled, rate of open arm entries, time spent in the open arms, time spent in the closed arms, and time spent in the center area (*n* = 17–29 per group). Data are presented as mean ± SD.

To assess locomotor activity and anxiety‐like behavior, we performed open field and elevated plus maze tests after the 1‐ or 3‐month intervention. In the open field test, mice habituated over the session, and total distance traveled did not differ between groups in either cohort, indicating that gross locomotor activity was unaffected by MG (Figure 3a). Time spent in the center was likewise comparable between groups, providing no evidence of altered anxiety‐like behavior. Consistent with this, in the elevated plus maze the time spent in the open arms, the principal index of anxiety‐like behavior, was not affected by MG in either cohort (Figure 3b). Among the secondary measures, the rate of entries into the open arms showed a significant treatment × period interaction, indicating opposite directional shifts at the two time points. In the 1‐month cohort, MG treatment significantly increased center area time, with smaller increases in the open arm entry rate and reductions in closed‐arm time that did not reach statistical significance, whereas in the 3‐month cohort MG reduced the open arm entry rate, a difference that did not reach statistical significance. These secondary changes were small and inconsistent between the two interventions.

### 3.4 MG mildly impaired hippocampus‐dependent spatial memory

**FIGURE 4:**
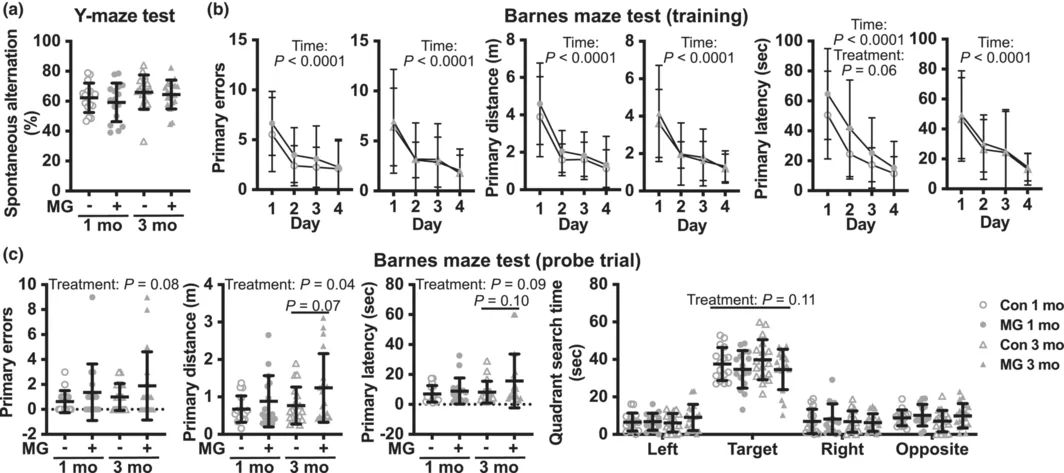
Y‐maze and Barnes maze performance in MG‐treated mice. (a) Spontaneous alternation in the Y‐maze test (*n* = 16–18 per group). (b) Primary errors, primary distance, and primary latency during Barnes maze training in the 1‐month and 3‐month interventions (*n* = 16–18 per group). (c) Primary errors, primary distance, primary latency, and time spent in each quadrant during the Barnes maze probe trial (*n* = 16–18 per group). Data are presented as mean ± SD.

Spontaneous alternation in the Y‐maze did not differ between groups (Figure 4a). During Barnes maze training, all groups improved across days in primary errors, primary distance, and primary latency, indicating intact task acquisition; a between‐group difference in primary latency that did not reach statistical significance was observed in the 1‐month intervention (Figure 4b). In the probe trial, MG significantly increased primary distance, with concomitant increases in primary errors and primary latency that did not reach statistical significance. Consistent with this, time spent in the target quadrant showed a reduction with MG that did not reach statistical significance, although all groups still searched preferentially in the target quadrant (Figure 4c). Together, these results indicate that MG mildly impaired hippocampus‐dependent spatial memory in the probe trial.

### 3.5 MG decreased excitatory synaptic gene expression in the hippocampus but not the prefrontal cortex

**FIGURE 5:**
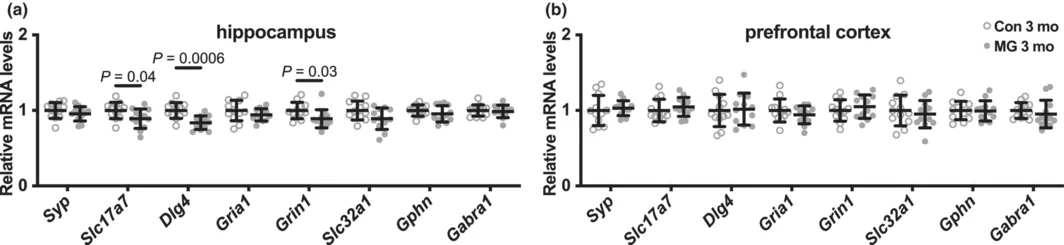
Synaptic gene expression in the hippocampus and prefrontal cortex. Relative mRNA expression of synaptic genes in the (a) hippocampus and (b) prefrontal cortex after the 3‐month intervention (*n* = 12 per group). Data are presented as mean ± SD.

We next examined the mRNA expression of synaptic genes in the hippocampus and prefrontal cortex after the 3‐month intervention. In the hippocampus, MG significantly reduced the expression of the excitatory synaptic genes *Slc17a7*, *Dlg4*, and *Grin1*, which encode VGLUT1, PSD‐95, and GluN1, respectively. In contrast, the pan‐synaptic marker *Syp*, the AMPA‐receptor subunit gene *Gria1*, and the inhibitory synaptic genes *Slc32a1*, *Gphn*, and *Gabra1* were unchanged (Figure 5a). In the prefrontal cortex, none of these genes was altered by MG (Figure 5b). Thus, after 3 months of MG exposure, the transcript levels of excitatory synaptic genes were selectively decreased in the hippocampus.

## 4 DISCUSSION

In this study, chronic oral administration of MG for 1 or 3 months produced partial metabolic alterations, mildly impaired hippocampus‐dependent spatial memory, and selectively reduced a subset of excitatory synaptic genes in the hippocampus. Notably, these synaptic and cognitive changes occurred without sustained fasting hyperglycemia, although MG induced clear insulin resistance and mildly impaired glucose clearance, and the gene‐expression changes were confined to the hippocampus and were not detected in the prefrontal cortex. We previously reported that type 1 diabetic mice show decreased expression of synaptic molecules in both the hippocampus and cortex (Yokokawa et al., 2023). The present findings indicate that MG exposure alone recapitulated part of these synaptic alterations, supporting the view that MG is a candidate factor contributing to the dysregulation of learning‐relevant excitatory synaptic genes in the hippocampus under diabetic conditions.

Previous studies linking MG to learning and emotional behavior demonstrate inconsistent results (Berends et al., 2025; Chun et al., 2016; Hambsch et al., 2010; Tsai et al., 2026). This inconsistency may be due to differences in the degree of MG burden across studies, owing to differences in the route, dose, and duration of administration. A strength of the present study is that we assessed metabolic phenotypes alongside the behavioral analyses under the same MG exposure paradigm, allowing us to evaluate comprehensively whether MG was biologically active. In this study, chronic MG treatment mildly impaired glucose clearance and induced insulin resistance, consistent with previous reports (Dhar et al., 2011; Guo et al., 2009). Notably, this mild glycemic dysregulation occurred without basal hyperglycemia or increased adiposity in the present paradigm. Indirect calorimetry further showed a lower RER, indicating a shift in metabolic substrate toward lipid oxidation, which indirectly suggests that this MG treatment paradigm impaired glucose metabolism. In a previous study, 50 mM MG in the drinking water has been reported to raise plasma MG approximately two‐fold, to a level comparable to that seen in type 2 diabetes (Berends et al., 2025), and the concentration used in the present study (1% w/v, approximately 139 mM) was substantially higher. This suggests that the present paradigm produced a circulating MG burden at least comparable to that of the diabetic state, although direct measurement in future studies would allow a more precise comparison. Together, the metabolic disturbances observed in this study indicate that the MG treatment was sufficient to impair glucose metabolism in the absence of obesity or hyperglycemia.

The metabolic effects of MG showed some dependence on exposure duration. Insulin resistance and the reduction in RER were already evident after the 1‐month intervention, indicating that impaired insulin action and a shift in substrate utilization are relatively early responses to MG. Impaired glucose clearance in the IPGTT was present at both time points; the difference reached statistical significance at the 30‐min post‐injection time point after the 1‐month intervention and in the overall area under the curve after the 3‐month intervention, indicating a slight but directionally consistent effect rather than a qualitatively different response. In contrast, the reductions in adiposity and total cholesterol emerged only after the 3‐month intervention, suggesting that changes in body composition require more prolonged MG exposure to develop.

Previous studies reported that intracerebroventricular and intraperitoneal MG treatment suppressed anxiety‐like behavior (Hambsch et al., 2010; Hansen et al., 2016; McMurray et al., 2016). Mechanistically, MG acts as a GABAA receptor agonist (Distler et al., 2012). On the other hand, oral MG administration was reported to exacerbate anxiety‐like behavior (Tsai et al., 2026), although another study failed to detect its effect (Berends et al., 2025), suggesting that the effect of oral MG administration on anxiety‐like behavior remains controversial. To address this, we performed open field and elevated plus maze tests, both of which are well‐established measures of anxiety‐like behavior. The principal indices of anxiety‐like behavior, center time in the open field test and open arm time in the elevated plus maze, were not affected by MG in either intervention. Although some secondary measures differed between groups, these changes were small and were not consistent between the 1‐ and 3‐month interventions. We therefore do not interpret the present data as evidence for a robust effect of oral MG on anxiety‐like behavior, and further studies using higher doses or alternative routes of administration, together with additional behavioral paradigms, will be needed to clarify whether MG modulates anxiety‐like behavior.

Regarding learning and memory, oral MG treatment mildly impaired performance in the Barnes maze test, whereas working memory in the Y‐maze test was unaffected. The effect on spatial memory was limited in magnitude. Although the measures showing a between‐group difference varied between the 1‐ and 3‐month interventions, these differences were small, and we interpret this variability as reflecting limited statistical power to detect a small effect rather than qualitatively distinct effects at the two time points. This mild impairment of spatial memory is nonetheless in line with previous reports: oral administration of 1% MG in drinking water has been reported to impair memory retention in the passive avoidance test (Chun et al., 2016) and oral MG impaired spatial learning and memory in the Morris water maze test (Tsai et al., 2026). In the former study (Chun et al., 2016), this impairment was observed at 1% (approximately 139 mM) but not at 0.5% MG, indicating a dose‐dependent effect. In contrast, a study using a lower concentration of MG (50 mM) in drinking water found no effect on spatial learning and memory in the Barnes maze test, even after prolonged exposure (Berends et al., 2025). Those authors themselves attributed the absence of a cognitive effect partly to their relatively low MG dose. Because all of these studies used oral administration, this discrepancy is unlikely to reflect differences in the route of administration and is instead consistent with a dose dependence. Together, these findings are consistent with a dose‐dependent action of oral MG on learning and memory, with the present 1% exposure being sufficient to elicit a mild deficit. However, because this action was limited in magnitude, studies employing higher doses or alternative routes of administration would help to determine the robustness and magnitude of this effect.

Synaptic plasticity in the hippocampus and PFC underlies cognitive and emotional function (Bannerman et al., 2014; Neves et al., 2008). In the present study, we examined the effect of MG on the expression of synaptic genes and found that MG decreased the transcript levels of the excitatory synaptic genes *Slc17a7*, *Dlg4*, and *Grin1* in the hippocampus but not in the PFC. These genes encode main components of hippocampal excitatory synapses—the presynaptic glutamate transporter VGLUT1, the postsynaptic scaffold PSD‐95, and the obligatory NMDA receptor subunit GluN1—whose functional loss or reduced expression impairs hippocampal excitatory synaptic transmission and plasticity and is associated with deficits in hippocampus‐dependent learning and memory (King et al., 2014; Migaud et al., 1998; Tsien et al., 1996). This hippocampal pattern partly corresponds to our previous observations in a type 1 diabetic mouse model, in which synaptic proteins such as GluN1 and VGLUT1 were broadly reduced (Yokokawa et al., 2023). In contrast, Dlg4 was reduced by MG treatment, whereas hippocampal PSD‐95 protein was not decreased in diabetic mice. This inconsistency may reflect differences between transcript and protein regulation, or between the effect of MG alone and that of the broader diabetic milieu. Therefore, although further investigation is required to uncover the precise underlying regulatory mechanism, these findings suggest that MG may contribute to impaired learning and memory by reducing the expression of genes involved in hippocampal neuroplasticity.

A limitation of this study is that only male mice were examined. Given that sex differences exist in metabolic and behavioral responses, the effects of MG may differ in females, and further studies including both sexes are warranted. In addition, the changes in synaptic gene expression are associational and do not establish a causal link to the behavioral alterations; thus, interventions that directly manipulate these gene expression changes in vivo will be required to test whether they mediate the cognitive effects of MG.

In conclusion, chronic oral MG administration impaired glycemic control without sustained fasting hyperglycemia, mildly impaired spatial memory, and selectively reduced the expression of excitatory synaptic genes in the hippocampus but not in the PFC, whereas it did not consistently affect anxiety‐like behavior. These hippocampal changes recapitulated part of the synaptic alterations previously observed in a diabetic mouse model, supporting the view that MG is a candidate factor contributing to the dysregulation of learning‐relevant excitatory synaptic genes under diabetic conditions. The mechanisms by which MG selectively affects hippocampal excitatory synapses, and the basis for this regional vulnerability, remain to be determined and warrant further study. These findings provide a basis for further studies on the mechanisms underlying diabetes‐associated cognitive impairment and on MG as a potential preventive or therapeutic target.

## AUTHOR CONTRIBUTIONS

**Akane Nakao:** Data curation; formal analysis; investigation; visualization. **Takumi Yokokawa:** Conceptualization; data curation; formal analysis; funding acquisition; investigation; methodology; project administration; visualization. **Fuminori Okazaki:** Investigation. **Nobumasa Iwanaka:** Funding acquisition; supervision. **Kazuo Inoue:** Funding acquisition; supervision. **Tatsuya Hayashi:** Funding acquisition; supervision. **Tatsuro Egawa:** Conceptualization; funding acquisition; supervision.

## FUNDING INFORMATION

This work was supported by the Japan Society for the Promotion of Science Grants‐in‐Aid for Scientific Research (No. 23K16695 and 25K03007 to T.Y., 24K14549 to N.I., 24K02881 to K.I., 23K27973 to T.H., and 25K02992 to E.T.).

## CONFLICT OF INTEREST STATEMENT

The authors declare no conflicts of interest, financial or otherwise.

## ETHICS STATEMENT

Animal experiments were approved by the Departmental Committee of Animal Experimentation at Kyoto University (Approval Number R8‐6A) and conducted in accordance with the Regulations on Animal Experimentation at Kyoto University.

## Data Availability

The data that support the findings of this study are available from the corresponding author upon reasonable request.
